# Uterine Carcinosarcoma: A Case Report and Literature Review

**DOI:** 10.1155/2020/8816348

**Published:** 2020-08-20

**Authors:** Ali Kord, Behnam Rabiee, Ismail Elbaz Younes, Karen L. Xie

**Affiliations:** ^1^Division of Interventional Radiology, University of Illinois College of Medicine, Chicago, IL, USA; ^2^Department of Radiology, University of Illinois College of Medicine, Chicago, IL, USA; ^3^Department of Pathology, University of Illinois College of Medicine, Chicago, IL, USA

## Abstract

Uterine carcinosarcomas are rare and extremely aggressive undifferentiated carcinomas which include both carcinomatous and sarcomatous elements. A 52-year-old female presented with heavy irregular menstrual bleeding for several years and new right elbow pain and swelling. Ultrasound and computed tomography showed a large uterine mass with regional and distant metastatic lymphadenopathy and suspicious findings of osseous metastasis to the right elbow. A biopsy confirmed uterine carcinosarcoma, and the patient underwent chemotherapy and then surgical resection of the uterine mass with palliative radiotherapy of the right elbow. The postoperative imaging showed new metastasis, and the patient was scheduled to start on immunotherapy. Considering the highly invasive nature of uterine carcinosarcomas, timely detection of this cancer using characteristic imaging and pathology findings is of extreme importance to improve the patient's survival.

## 1. Introduction

Uterine carcinosarcomas are rare tumors comprising less than 5% of uterine malignancies [1, 2]. Uterine carcinosarcomas are extremely aggressive undifferentiated carcinomas which include both carcinomatous and sarcomatous elements, arising from a single malignant epithelial clone [2–6]. The prognosis is often poor, with 30–40% of cases having extrauterine involvement at the first presentation [7]. The clinical presentation of the uterine carcinosarcomas is nonspecific, and imaging and pathology studies play an important role in diagnosis [7]. In this study, we present a rare case of uterine carcinosarcoma with a concise review of the most prominent radiologic and histologic characteristics of this rare entity.

## 2. Case Report

A 52-year-old G4P3 female presented to the gynecology outpatient clinic with complaints of heavy irregular menstrual bleeding for several years and new right elbow pain and swelling. The patient denied any unintentional weight loss, lightheadedness, palpitation, shortness of breath, urinary symptoms, or difficult defecation. The pelvic exam demonstrated normal external genitalia with a large lower uterine-cervix mass. The physical examination was remarkable for an enlarged firm right supraclavicular lymph node. The right elbow appeared swollen with a severely compromised range of motion due to pain. The patient underwent an exam under anesthesia with a biopsy of the uterine mass.

**Figure 1:**
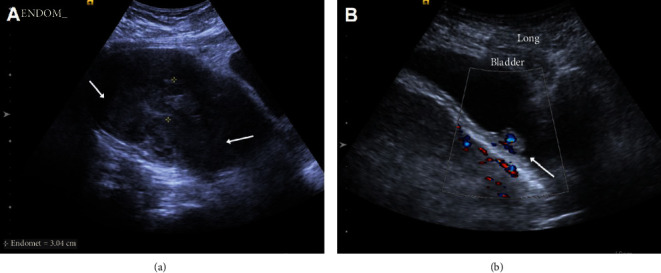
Grayscale ultrasound image of the uterus (a) demonstrates a heterogeneous uterine echotexture with poorly defined masses (arrow, (a)). The endometrial stripe is thickened (clippers, (a)) and measures up to 3.0 cm. Color Doppler image (b) shows a soft tissue mass within the posterior wall of the urinary bladder (arrow, (b)) with vascularity.

**Figure 2:**
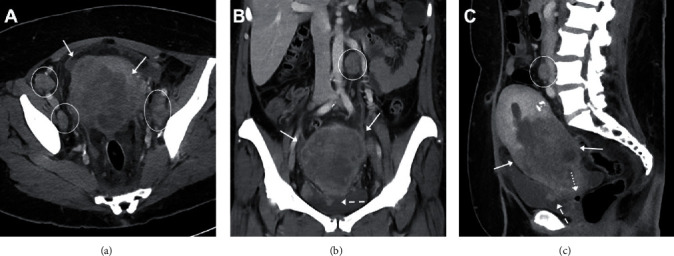
Axial (a), coronal (b), and sagittal (c) postcontrast CT images show a large heterogeneous mass centered within the lower uterus and cervix (solid arrows) which involves more than 50% of the myometrium and is enhancing less than the normal myometrium. There is local invasion to the urinary bladder (dotted arrows, (b, c)) and bilateral pelvic and retroperitoneal lymphadenopathy (circles/ellipses, (a–c)). There is no clear distinction with loss of the fat plane between the vagina and the rectum. Air focus within the vagina (dashed arrow, (c)) is suspicious for a rectovaginal fistula.

**Figure 3:**
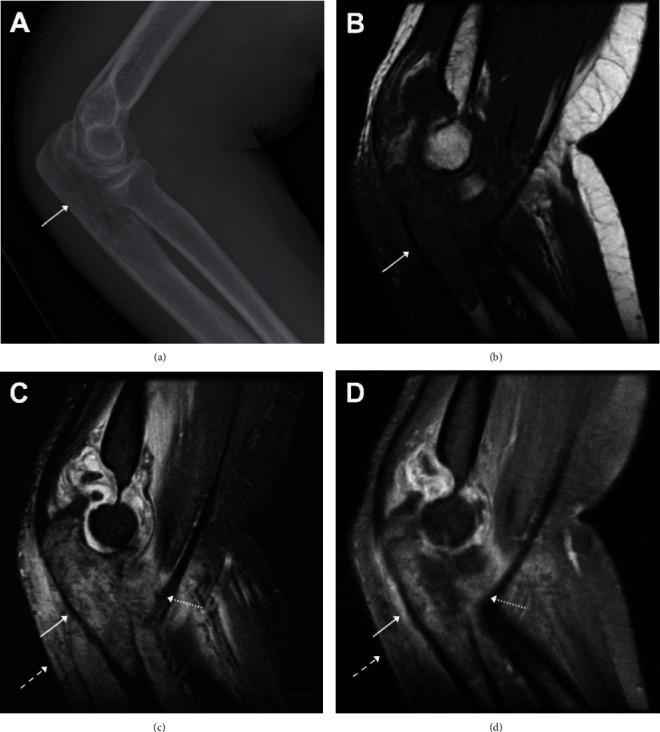
Lateral right elbow radiograph (a) shows a lytic lesion within the proximal ulna with permeative or moth-eaten appearance and wide zone of transition, suggestive of an aggressive lesion. Sagittal noncontrast T1-weighted (b), fluid-sensitive (c), and postcontrast (d) MRI images demonstrate an infiltrative mass centered within the proximal ulna (solid arrows, (b–d)) with soft tissue invasion (dotted arrow, (c, d)). There is replaced normal bone marrow signal (b), increased T2 signal (c), and postcontrast enhancement (d). The adjacent muscle demonstrates increased signal on the fluid-sensitive image (dashed arrow, (c)) with mild enhancement (dashed arrow, (d)), due to soft tissue invasion.

Ultrasound of the pelvis (Figure 1) showed heterogeneous echotexture of the uterus with possible masses, a thickened endometrial stripe, and a vascular soft tissue lesion within the posterior wall of the urinary bladder. Computed tomography (CT) of the chest, abdomen, and pelvis (Figure 2) demonstrated a large heterogeneous mass centered within the uterus and cervix which involved more than 50% of the myometrium. There was local invasion to the urinary bladder, bilateral pelvic and retroperitoneal lymphadenopathy, and suspicious findings for a rectovaginal fistula. A right supraclavicular lymph node was found, and the patient underwent ultrasound-guided biopsy of the lymph node. A right elbow radiograph showed findings concerning an aggressive lytic lesion within the proximal ulna (Figure 3). Magnetic resonance imaging (MRI) of the right elbow (Figure 3) confirmed an enhancing mass centered within the proximal ulna with soft tissue invasion.

**Figure 4:**
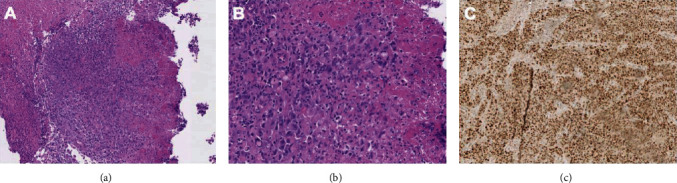
(a) Low power: 80x magnification showing replacement of the normal endometrium by malignant cells with necrosis. (b) High power: 200x magnification showing pleomorphic malignant cells effacing the normal endometrial tissue. (c) p53 staining: 100x magnification showing positive staining for p53 in malignant cells.

The pathology report from the cervical biopsy (Figure 4) revealed infiltrating malignant high-grade carcinoma with areas of undifferentiated sarcomatous morphology, which was positive for vimentin. The mutation pattern studied by molecular pathology study suggested an endometrioid differentiation with ARID1A mutation and lack of p53 mutation. The aggressive behavior of the tumor and morphologic findings were highly suggestive of carcinosarcoma. Biopsy of the right supraclavicular lymph node revealed malignant cells with similar features to the uterine lesion which were positive for cytokeratin 7 and negative for cytokeratin 20. The biopsy of the right elbow masses was negative for malignancy, but it was considered a false negative due to inadequate sampling and given its aggressive imaging findings and evidence of other distant metastasis.

The patient was started on a monthly carboplatin and paclitaxel chemotherapy regimen based on National Comprehensive Cancer Network (NCCN) guidelines for 6 cycles [8]. Palliative radiation was started for the suspicious metastasis of the right elbow, as well as denosumab to decrease fracture risk due to bony metastasis. The patient was followed regularly as an outpatient, and at the last follow-up visit six months after starting the chemotherapy and completing the right elbow radiotherapy, the patient complained of minimal vaginal spotting. There was significant relief in the right elbow pain with return to almost normal function. The patient finally underwent total hysterectomy and bilateral salpingo-oophorectomy, omentectomy, and pelvic and para-aortic lymphadenectomy. The final surgical pathology study confirmed the diagnosis of carcinosarcoma with components of poorly differentiated endometrioid carcinoma and sarcomatous changes. The sarcomatous component stained positive for desmin and vimentin and was negative for pancytokeratin. The tumor cells did not show loss of BRG1 (SMARCA4) immunohistochemical stain, supporting the above diagnosis [9]. The follow-up postoperative positron emission tomography-CT (PET-CT) showed new metastases in the head, chest, and abdomen, and the decision was made to start the patient on immunotherapy by pembrolizumab and lenvatinib.

## 3. Discussion

### 3.1. Epidemiology and Clinical Presentation

The incidence of uterine carcinosarcoma in the United States is approximately 1 to 4 per 100,000 women [10], with a peak incidence in the age range 62-67 years [11]. African Americans are twice at risk compared to Caucasians [12, 13]. Other risk factors include obesity, nulliparity, exogenous estrogen, tamoxifen, and exposure to pelvic radiation, while progestin-containing contraceptives are thought to have protective effects [2, 14]. In addition, the association between smoking and tumor aggressiveness and increased risk of uterine sarcoma has been reported [15–17].

Clinical presentation is often the triad of abnormal uterine bleeding, pain, and a rapid uterus enlargement [7]. In physical examination, the tumor presents as a relatively large polypoid mass which could protrude from the cervix, posing the risk of uterus inversion [7]. Uterine carcinosarcomas have high rates of lymphatic spread, peritoneal seeding, and pulmonary metastasis [18]. Approximately 30–40% of cases have extrauterine involvement at the first presentation, extrauterine nodal spread being widely prevalent, and over 10% of patients initially present with distant metastasis [7]. These contribute to the poor prognosis of this rare disease. Differential diagnoses consist of endometrial carcinomas (e.g., endometrioid, serous, clear cell, and dedifferentiated carcinomas), as well as mesenchymal malignancies of the uterus (e.g., adenosarcoma, leiomyosarcoma, and undifferentiated uterine sarcoma) [7].

### 3.2. Genetics and Pathology Findings

Uterine carcinosarcoma is most commonly of uterus origin, but it can rarely arise from the fallopian tube, cervix, or peritoneum [7, 19]. On immunohistological analysis, uterine carcinosarcomas demonstrate both epithelial and stromal markers including p53, vimentin, CD10, smooth muscle actin (SMA), and desmin [2]. The carcinoma portion of the carcinosarcomas can be serous or endometrioid in origin with endometrioid being much less frequent. The lack of p53 mutation is against serous, and the presence of ARID1A mutations is suggestive of endometrioid carcinomas, although only seen in up to 20% of carcinosarcomas [2, 12]. Commonly associated mutations include *TP53* mutations (91%) and PI_3_K pathway genes (50%, specifically PIK_3_CA comprising 35%), providing potential targets for therapies [5]. Other genetic associations include mutations in *PTEN*, *KRAS*, *PPP_2_R_1_A*, *CHD_4_*, and *BCOR*, as well as abnormalities in histone H2A/H2B expression [7].

### 3.3. Imaging Findings

Imaging is a major part of the pre- and posttreatment evaluation of uterine carcinosarcoma, with highlighted roles in surgical planning and adjuvant therapies [2, 7, 19]. Sonography is commonly used as an initial assessment in the suspected cases with uterine abnormality. Early stages of uterine carcinosarcomas present on ultrasound as hyperechoic masses relative to the endometrium and may show a thickened heterogeneous endometrial stripe with expansion of the endometrial canal [7]. CT is the preferred modality for staging, follow-up, and evaluation of distant metastases [2, 7, 8]. Compared to other endometrial carcinomas, early stages of carcinosarcomas may appear as heterogeneous masses with a greater craniocaudal width [7, 19]. On CT, the tumors present as ill-defined hypodense mass(es) with dilation of the endometrial cavity in most cases [7]. Uterine carcinosarcoma can metastasize to a wide variety of organs, including the lungs (49%), peritoneum (44%), bones (17%), and liver (15%), as well as the central nervous system (8%), with the latest being associated with poor prognosis [20, 21]. Staging of the endometrial cancers is summarized in Table 1 [22].

**Figure 5:**
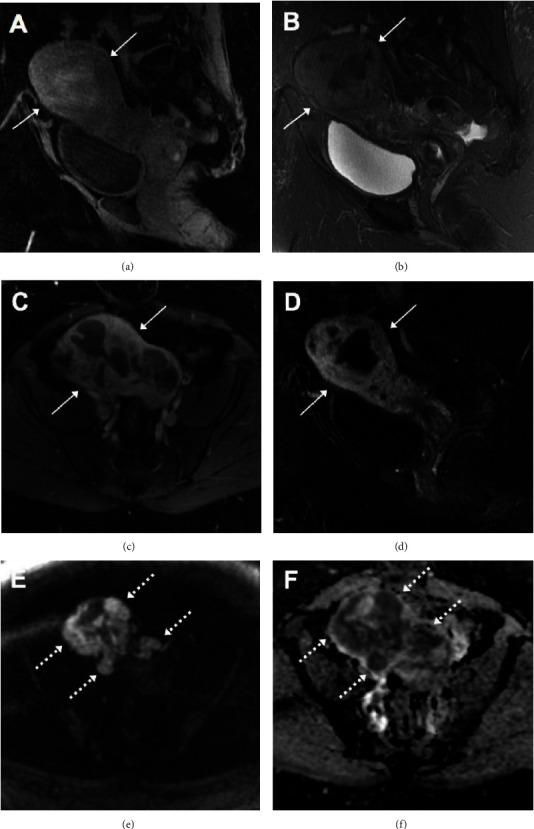
An example of pelvic MRI in a patient with high-grade uterine sarcoma (not the case presented in this study). The sagittal precontrast T1-weighted (a) and T2-weighted (b) MRI images demonstrate a heterogeneous mass (solid arrows, (a–d)) centered within the uterus which demonstrates enhancement on postcontrast axial (c) and sagittal (d) T1-weighted fat-saturated images. There are areas with restricted diffusion within the mass (dotted arrows, (e, f)) demonstrating high signal on DWI (e) with corresponding low signal on the ADC map (f).

MRI, specifically T2-weighted images (T2WI), is depicting an excellent contrast of uterine anatomy and is superior in imaging of uterus malignancies. An example of uterine MRI in a patient with a high-grade uterine sarcoma is shown in Figure 5. The areas of sarcomatous differentiation may show early persisting enhancement which may distinguish uterine carcinosarcoma from endometrial adenocarcinoma [7]. On T1-weighted images (T1WI), endometrial adenocarcinomas are usually isointense relative to the normal endometrium and are hypointense to the endometrium on T2WI [7, 23]. On postcontrast T1WI, uterine carcinosarcomas demonstrate slower enhancement relative to the surrounding myometrium. In addition, carcinosarcomas, similar to other endometrial tumors, show high signal intensity on diffusion-weighted imaging (DWI) and low signal intensity on apparent diffusion coefficient (ADC) maps [7, 24].

Minimally invasive biopsy of nodal disease is proven to be beneficial in different cancer types, especially considering the high morbidity associated with total lymphadenectomy [7]. MRI and fluorodeoxyglucose (FDG) PET-CT can help detect lymph node involvement prior to surgery [25]. Specifically, one study investigated FDG PET-CT and sentinel lymph node mapping and demonstrated that progression-free survival can be maintained with minimally invasive sentinel lymph node mapping, with no significant difference in outcome compared to total lymphadenectomy [26]. FDG PET-CT was found to be more sensitive but less specific compared to MRI at detecting nodal metastasis (sensitivity 68% versus 50% and specificity 88% versus 93%, respectively) [25]. However, there is still a considerable risk of missing micrometastatic disease by imaging due to the limited sensitivity, and lymphadenectomy is considered the gold standard for nodal assessment [25]. Despite these limitations, FDG PET-CT could be beneficial for initial staging [7].

In the past decade, PET-MRI has been evolving as a capable modality for endometrial cancer evaluation [25, 27]. While PET-CT is generally superior for detecting distant metastasis, MRI can better demarcate pelvic organs' anatomy and plays an essential role in evaluating the extent of local involvement [27]. PET-MRI has shown better performance in the detection and evaluation of the extent of primary endometrial tumors, with comparable accuracy for the detection of pelvic lymph node metastases compared to PET-CT [28].

### 3.4. Treatment and Outcome

Given the aggressive nature of uterine carcinosarcoma compared to other conditions in the differential diagnosis, making an early and correct diagnosis and choosing the correct treatment approach are of extreme clinical importance. Treatments of uterine carcinosarcoma consist of surgery, radiotherapy, and chemotherapy. The rarity of the disease is a challenge in performing large-scale randomized clinical trials to properly assess the treatments with significant outcomes [7]. Total hysterectomy and bilateral salpingo-oophorectomy along with surgical staging are the first line in patients without distant metastasis [8]. Adjuvant chemotherapy was found to be more effective compared to radiotherapy in some studies [7]. The local recurrence may be treated with radiotherapy or systemic chemotherapy [8]. Single-metastatic brain metastasis is managed with surgical removal of the tumor or stereotactic radiosurgery, after which adjuvant whole-brain radiotherapy is performed [29]. Palliative treatment with steroids and whole-brain radiation are considered for cases with multiple brain metastases [29].

The patients with uterine carcinosarcomas should be closely followed up regardless of the disease state, because there is a high risk of local recurrence (60%) and distant metastasis [2]. The risk of recurrence is higher with larger tumors, higher cancer stage at initial presentation, the existence of lymphovascular involvement, and deeper myometrial invasion [7]. Clinical follow-up is usually performed with a physical exam, and vaginal cytology is recommended every 3 months for 2 years, then every 6 months for 5 years [7].

The NCCN guidelines recommend baseline imaging as well as follow-up imaging to detect metastatic disease, especially considering the high recurrence rate [8]. Abdominal-pelvic or chest CT is recommended for cases with clinical concern for recurrence or metastasis. Whole-body PET-CT should be offered to patients who are being considered for locoregional therapy or surgery [8]. Following initial treatment of cases with late stages (III/IV) of uterine carcinosarcoma, a full-body or pelvic CT scan is recommended every 6 months up to 3 years and then every 6–12 months for another 2 years [7].

The overall prognosis of uterine carcinosarcoma is poor, even with the best of care, due to its aggressive behavior [2, 14]. The surgical stage is the most important prognostic factor, and deep myometrial invasion and extrauterine extension predict poor prognosis [12]. The 5-year survival is 60-75% for uterine-confined disease, 40-60% for early-stage disease (I and II), and 15-30% for late-stage disease, with overall median survival of less than 2 years [12].

## 4. Conclusion

In summary, a rare case of uterine carcinosarcoma is reported in this study. Considering the highly invasive nature of uterine carcinosarcomas, timely detection of this cancer using characteristic imaging and pathology findings is of extreme importance to improve the patient's survival.

## Figures and Tables

**Table 1:** Staging of the endometrial cancers [22].

Stage	Description	Subgroups
Stage I	Tumors are confined to the corpus uteri	IA: <50% myometrial invasion IB: ≥50% myometrial invasion
Stage II	Tumors invade the cervical stroma but do not extend beyond the uterus	
Stage III	Local and/or regional spread of the tumor	IIIA: serosa and/or adnexa involvement IIIB: vaginal and/or parametrial involvement IIIC1: pelvic lymph node involvement IIIC2: para-aortic lymph node involvement
Stage IV	Invasion to the bladder and/or bowel mucosa and/or distant metastasis	IVA: invasion of the bladder and/or bowel mucosa IVB: intra-abdominal metastases and/or inguinal lymph node metastasis

## Data Availability

The clinical data used to support the findings of this study are restricted by the UI Health IRB in order to protect patient privacy. Data are available from corresponding author (KX) for researchers who meet the criteria for access to confidential data.
